# Psychological Associations of Stress with the Level of Health Locus of Control and Self-Efficacy in Patients with Ovarian Cancer

**DOI:** 10.3390/jcm12216816

**Published:** 2023-10-28

**Authors:** Edyta Skwirczyńska, Aneta Cymbaluk-Płoska, Oskar Wróblewski

**Affiliations:** 1 Department of the History of Medicine and Medical Ethics, Pomeranian Medical University, Rybacka 1, 70-204 Szczecin, Poland; 2 Clinical Department of Reconstructive and Oncological Gynecology, Pomeranian Medical University, 70-204 Szczecin, Poland; aneta.cymbaluk@gmail.com (A.C.-P.); oskraw@gmail.com (O.W.)

**Keywords:** ovarian cancer, coping mechanisms, locus of control, self-efficacy, psycho-oncology

## Abstract

The aim of this study was to analyze the locus of health control, self-efficacy and stress coping styles of female patients treated for ovarian cancer. Learning the styles of coping with stress in patients with ovarian cancer may contribute to improve their quality of life after cancer diagnosis. A series of Pearson’s r-analyses was performed in the order to evaluate the hypotheses regarding the relationship between styles of coping with stress, the locus of health control and self-efficacy. A total of 151 female patients participated in this study. Standardized psychological questionnaires were used: the General Self-Efficacy Scale (GSES) to measure coping with difficult situations and obstacles, the Multi-Dimensional Health Locus of Control Scale (MHLC) to measure health control and the Convergence Insufficiency Symptom Survey (CISS) to measure stress coping styles. All questionnaires had an adaptation in Polish. Patients using task-focused and socializing styles had higher self-efficacy, whereas focusing on negative emotions resulted in lower self-efficacy. External locus of health control was related to a task-focused approach to treatment. On the other hand, the focus on negative emotions was related to the feeling that the fate of patients was decided by chance. Self-efficacy was positively associated with internal locus of health control and with external control, which means the influence of others. The results of our study indicate the need for a multidimensional approach to the treatment of female patients with ovarian cancer. The psychological condition of female patients has an ongoing relationship with their physical health.

## 1. Introduction

In developing countries, ovarian cancer is the fourth most common cause of death among women. Late detection is resulting in extensive abdominal metastases and is mainly responsible for the high mortality rate. Nowadays, modern treatment methods guarantee a complete response in the majority of treated patients. However, these methods do not protect against disease recurrence in the case of high-grade disease, which occurs in patients after an average of 18 months [1]. If the cancer is detected late, the chances of 5-year survival for patients are reduced from 45% to 25% [2].

Coping strategies play a crucial role in the life of the patient who suffers from an illness, such as cancer. One of the factors that improves the ability to cope with a cancer diagnosis is a sense of self-efficacy [3]. Given the frequent recurrence in patients with advanced-stage ovarian cancer, it becomes extremely important that patients receive appropriate psychological care. Patients’ quality of life depends not only on the treatment process but on physiological factors too. Psychological factors also have a major impact on the development of quality of life. Studies show that among female cancer survivors, fear of cancer progression affects between 22 and 99% [4]. Patients with ovarian cancer are at constant risk of experiencing negative emotional states, anxiety and depression. Chronic stress may contribute to biological responses leading to the process of carcinogenesis [5]. In patients diagnosed with cancer, anxiety is related to cancer progression indicated as one of the main factors causing anxiety symptoms [6].

One of the key factors during cancer treatment is self-efficacy. According to Bandura’s social-cognitive theory, self-efficacy determines the amount of effort a person will put in during a difficult situation and how long they are able to maintain it despite unfavorable circumstances [7]. It has been shown that a higher sense of self-efficacy can promote coping with stress, help control pain and have a mobilizing effect on the immune system [8]. Information about the disease elicits a range of reactions that depend on individual personality traits. Patients present two types of attitudes in response to the diagnosis—an active one, focused on the condition of their own health, and a passive one, resulting in anxiety and resignation [9]. Self-efficacy is related to the specific behaviors of patients who are at the various stages of dealing with cancer. The self-efficacy concept is limited compared to control, which encompasses the behaviors that lead to expected outcomes. According to Rotter’s social learning theory, individuals’ behaviors are largely predictable. Changes in the patients’ thoughts and the environment can lead to changes in their behavior. Therefore, patients’ actions are associated with their specific life experiences. Although, it is necessary to emphasize that such actions can be changed with the interventions in the different areas of the patient’s life [10,11,12]. Increased self-efficacy level leads to reduced depressive symptoms and better adaptability to the stressful situations. Among chronically ill patients with an internal sense of health control, there are notable feelings of helplessness and frustration due to a lack of control over health-related decisions. Paradoxically, an external control can contribute to better functioning during an illness. The patients are not obligated to constantly control their environment, which reduces the level of their frustration [13]. The assessment of the patient’s control over their health allows for the further prediction of their health-promoting and preventive behaviors and also influences their decision making during the treatment process [14]. It has been proven that the cancer patients who control their health and level of the stress can cope much better cognitively, emotionally and behaviorally. Increased self-efficacy level leads to reduced depressive symptoms and better adaptability to stressful situations [15,16]. An important psychological factor that can both positively and negatively influence the recovery process is patients’ sense of locus of control. It is classified as one of the personality dimensions. Patients with an external locus of control attribute importance to the situation as being beyond their control, whereas patients with an internal locus of control rely on their own decisiveness and the actions they take [17,18]. Studies have shown that the patients who have underwent dialysis and had more believed themselves to have more control proved to have an improvement in their quality of their life. They also experienced less depressive symptoms, which has led to a more effective treatment with reduced amount of adverse symptoms and complications [19].

In the face of illness, it is crucial to observe how individuals cope with stress. The choice of specific coping strategies can have a positive or a negative impact on how patients will adapt to information about their cancer. The concept of coping styles has been developed to address the various ways that individuals deal with stress [20]. Coping with stress plays a significant role in patients’ adaptation to illness. The coping process manifests itself as the ability to overcome stress factors and manage one’s own emotions [21,22]. A distinction is made between problem-focused strategies, which aim to solve a problem or change a stressor. On the other hand, emotion-focused involves reducing the emotional stress caused by the situation the individual is in [23]. It has been indicated that an external locus of control may exacerbate patients’ experience of pain. An external locus of control also contributes to the choice of maladaptive coping strategies and exacerbates the process of pain control [24]. It is important to control the level of the stress experienced by patients because it often coincides with cancer. A high stress level can contribute to tumor progression by activating the signal pathways in cancer cells [25]. Different coping strategies have been observed among cancer patients. Patients who have deep faith accept their illnesses easier by believing that their God has sent the disease in order to test them and has control over them [26]. In another study, differences in strategies were observed between breast cancer patients based on their ethnic backgrounds. White patients tended to use humor-based coping strategies, while Latino patients relied more on the emotion-focused strategies, and African–American patients focused on their religion and deep faith [27]. The diagnosis of cancer or illness can be the cause of a vast amount of stress. Cancer not only impacts biological functions but also one’s social and daily life. Furthermore, it is essential to adopt a multidimensional classification model proposed by WHO. The application of this model allows for a holistic perspective on disease in the biological, social and individual aspects. Numerous studies have proven that stress and a lack of the coping methods increase the likelihood of the development of the cancer. This also influences the chances of survival. Among patients who are unable to cope with the stress, the probability of survival is visibly reduced [28,29].

Considering the theoretical framework and reports presented above, the objective of this study is to analyze the locus of health control, self-efficacy and stress coping styles of female patients treated for ovarian cancer to verify the following hypotheses: (1) level of self-efficacy is positively correlated with the task-focused style; (2) level of internal locus of health control is positively correlated with the task-focused style; (3) level of the internal locus of control is positively correlated with the level of self-efficacy; (4) level of self-efficacy is negatively correlated with unadaptive styles; (5) level of internal locus of health control is negatively correlated with unadaptive styles; (6) the level of the external locus of control is negatively correlated with the level of self-efficacy.

## 2. Materials and Methods

We conducted a cross-sectional study to analyze stress coping styles, self-efficacy and the locus of health control. The study included 151 women treated for ovarian cancer at the Independent Public Clinical Hospital No. 2 of Pomeranian Medical University in Szczecin (SPSK2). For the purposes of this study, we assumed a minimum sample size of 150 patients. The study ran from January 2022 to February 2023. The inclusion criteria for the study were patients with suspected ovarian cancer after imaging studies, such as ultrasonography and computed tomography, and with histopathological confirmation of ovarian cancer. Criteria for exclusion from the study included: coexistence of other cancers, diagnosed endometriosis, coexistence of collagenosis, psychiatric treatment, psychological therapy before the diagnosis of ovarian lesions. Eligibility was based on biopsy results and diagnostic imaging. Polish adaptations of the questionnaires were used for the study since that all treated patients were of Polish nationality. The questionnaires were delivered to the patients by a psycho-oncologist while the patients were in hospital between chemotherapy cycles. Each patient was informed of the details of the study, as well as the possibility of withdrawing from it at any time. Patients completed questionnaires in their hospital rooms. Each given questionnaire was handed in an envelope and the psycho-oncologist was present with each patient throughout the questionnaire completion. After completion, patients were asked to put the questionnaires back in the envelope and to close it. Each study participant was additionally given an informed consent form. Lack of informed consent or failure to complete one of the questionnaires resulted in the patient being excluded from the Study. A total of 173 patients received the questionnaires, of which complete questionnaires with the informed consent statement were returned by 151 patients.

Patients were asked to fill the following questionnaires: a demographic data questionnaire, the General Self-Efficacy Scale (GSES) (Supplementary Materials, Questionnaire Q1), the Multidimensional Health Locus of Control (Supplementary Materials, Questionnaire Q2) and the Coping Inventory for Stressful Situations (CISS) (Supplementary Materials, Questionnaire Q3). The demographic questionnaire consisted of 3 questions about patients’ age, place of residence and education. The Generalized Self-Efficacy Scale (GSES) is a psychometric tool used to measure the level of generalized self-efficacy, understood as the belief in an individual’s ability to successfully cope with various life situations. The design of the tool consists of 10 questions about achieving goals, making decisions, overcoming difficulties or taking on new challenges. The questions were included on a 4-point Likert scale, where 0 indicates complete disagreement and 4 indicates complete agreement. The results obtained indicate the level of generalized efficacy of the respondents. The internal consistency of the tool was estimated based on a survey of 174 people: the Cronbach’s alpha coefficient was 0.85 and the reliability was 0.78. Due to its design, the questionnaire is applicable in scientific research and clinical practice, allowing for more precise intervention and psychological measures [30].

The Multidimensional Health Locus of Control Form A (MHLC) is a tool used to explore multidimensional perceptions of health control. It provides insight into an individual’s beliefs about how their health and illnesses are controlled. The questionnaire consists of 18 questions, each of which is rated on a 6-point scale, with 1 meaning that the surveyed person strongly disagrees and 6 meaning that the surveyed person strongly agrees. The design of the questionnaire is based on three dimensions: internal control, understood as the real influence on one’s health and illness; external control, where the person believes that their health is influenced by external factors such as fate, other people or genetics; and random control, where the person believes that their health is determined by chance and their decisions are irrelevant. The questionnaire is used in both clinical and research studies to explore people’s beliefs about health control and to better understand behaviors related to adherence to a treatment plan, maintaining a healthy lifestyle or working with a medical team. The Cronbach’s alpha coefficient for the A version is 0.74 for internal control, 0.69 for the case and 0.54 for the other [11].

The Coping Inventory for Stressful Situations (CISS) is a questionnaire used to measure coping styles in difficult situations. The design of the questionnaire is based on 48 questions on a 5-point Likert scale, where 1 means not true and 5 means completely true. The questions in the questionnaires concern different ways of coping with stress. The results of the questionnaire make it possible to distinguish three main coping styles, which include problem-focused coping, emotion-focused coping and avoidance coping. The questionnaire can be used in research on stress and in clinical practice, helping to provide well appropriate treatment for patients suffering from anxiety and depressive disorders. The survey has high accuracy and high internal consistency (0.78–0.90 in accordance with Cronbach’s alpha) [31].

## 3. Results

To verify the hypotheses, statistical analyses were performed by using the IBM SPSS Statistics 25 package, with frequency analyses, descriptive statistics analyses including the Kolmogorov–Smirnov test and Pearson’s r correlation analyses. The classic threshold of alpha = 0.05 was considered as the significance level. As a first step, the basic descriptive statistics of the quantitative variables under study were counted together with the Kolmogorov–Smirnov tests, which check the normality of the distribution of the variables under study. As can be seen in Table 1, the distributions of task-focused style, internal control scales and case version A were close to a normal distribution, as indicated by the statistically insignificant Kolmogorov–Smirnov test result. For the other variables, distributions different from the normal distribution were noted. In such a situation, it is advisable to additionally verify the skewness value of the distribution of the variables under study. If it is within ±2, it can be assumed that the distribution of the variable under study is not significantly asymmetric with respect to the mean [32]. Such a value has been reported for all variables under study. Therefore, in this chapter, statistical analyses will be performed using parametric tests.

### 3.1. Demographic Data

The study involved 151 women aged 34 to 83 (*M* = 62.06; *SD* = 9.86), 39.47% of the respondents had higher education, 47.11% had secondary education, and 13.42% had vocational education. A total of 44.8% of the respondents came from a city with more than 100,000 inhabitants, 38.05% came from smaller towns and the remaining respondents (17.15%) came from rural areas.

### 3.2. Level of Self-Efficacy vs. Level of Stress Coping Styles

**Figure 1:**
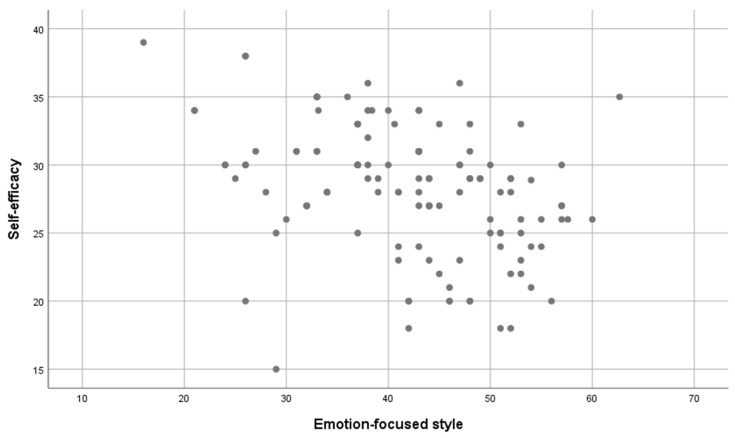
The level of task-focused style and the level of self-efficacy.

**Figure 2:**
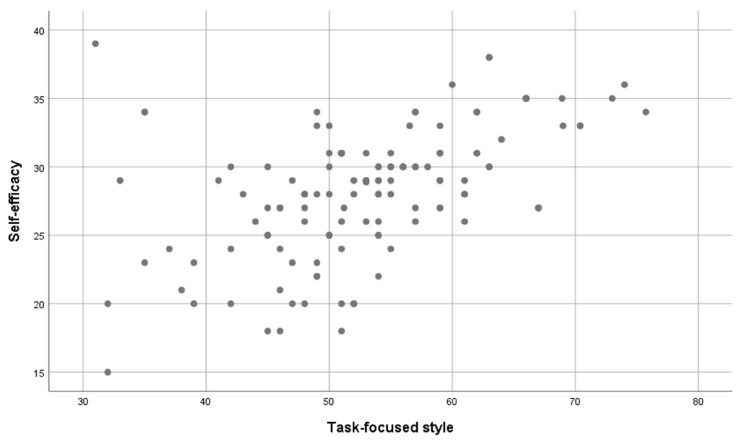
The level of style focused on emotions and the level of self-efficacy.

In the next step, it was examined whether the level of self-efficacy was correlated with the level of stress coping styles. A series of Pearson’s r correlation analyses was performed. Four statistically significant correlations are noted in Table 2. The level of self-efficacy was higher as the level of task-focused style increased (see Figure 1) and the socializing–seeking scale, and lower as the level of the emotion-focused style increased (see Figure 2) and the engaging in vicarious activities scale increased. The relationship between self-efficacy and task-focused style was strong, the relationship with emotion-focused style was of moderate strength, and the other two statistically significant relationships were weak. Only the correlation between self-efficacy and avoidance-focused style was not statistically significant.

### 3.3. Level of Locus of Control of Health versus Level of Stress Coping Styles

In the next step, it was examined whether the level of self-efficacy was correlated with the level of stress coping styles. A series of Pearson’s r correlation analyses was performed. First, variant A of the MHLC questionnaire was considered Table 3. Two statistically significant correlations were noted. The level of task-focused style correlated positively with the level of the others’ influence scale, and the level of emotion-focused style correlated positively with the level of emotion-focused style. Thus, the higher the level of these loci of health control scales, the higher the level of the indicated stress coping styles. The strength of both associations was low. The remaining correlations were not statistically significant.

### 3.4. Level of Locus of Control of Health versus Level of Self-Efficacy

In the next step, it was examined whether the level of the locus of health control was correlated with the level of self-efficacy. A series of Pearson’s r correlation analyses was performed. First, variant A of the MHLC questionnaire was considered Table 4. Two statistically significant correlations were noted. The level of self-efficacy was positively correlated with the level of the internal control scale and the influence of others. It means that the level of self-efficacy was higher as the the level of these two scales of locus of health control was higher. The strength of both relationships was low. The relationship between self-efficacy and the case scale was not statistically significant.

## 4. Discussion

Our results indicated that there was a strong relationship between self-efficacy and the level of task-focused strategies and social contact seeking. Patients focusing on problem identification and ways to solve the problem were characterized by higher self-efficacy. Self-efficacy decreased with increasing levels of emotion-focused strategies and engaging in vicarious activities. Focusing on emotions, often resulting in a focus on negative emotional experiences such as guilt, tension or anger resulted in lower self-efficacy in female patients. Similar to the work of Bakan and Inci, who showed that stress coping styles were a significant predictor of self-efficacy among patients with heart disease, stroke, cancer and chronic respiratory disease [33]. A meta-analysis by Chirico et al. showed that self-efficacy was helpful for coping strategies in cancer. The results indicate that a high level of self-efficacy contributes significantly in reducing distress [34]. In a study by Kreitler et al. self-efficacy was shown to be an important factor in regulating patients’ quality of life. High levels of self-efficacy not only contributed to the reduction of distress but had a positive impact on their quality of life [35]. Like our study, Amirshamsi et al. showed that self-efficacy decreased as the level of emotion-focused style increased [36]. Supporting patients to adopt task-focused and social-seeking styles may prove helpful in enhancing self-esteem. It has been indicated that intervention by a psycho-oncologist may help to reduce cortisol levels in patients diagnosed with cancer. Appropriate stress management following intervention by a psycho-oncologist and the provision of social support to patients has a beneficial effect on the course of cancer [37].

We have demonstrated that a task-focused style correlated with the external location of health scale. The external location of health control by individuals is considered to be a behavior that is not conducive to health and is also often a manifestation of a lack of responsibility for one’s own health. Surprisingly, patients presenting an external location of health control showed a correlation with a task-based style, which is characterised by a maladaptive approach to a stressful situation [38]. These results may be caused by the fact that patients, on the one hand, try to take a task-based approach to their illness and, on the other hand, are aware of the poor prognosis of ovarian cancer, which causes them to take actions that are not conducive to their own health. In our study, we also showed that an emotion-focused style correlated positively with the dimension of locus of external control that was chance. This may be because patients attributing the causation of their illness to chance are more bitter and therefore more likely to focus on experiencing negative emotions. Adequate psychological care that allows patients to work through their illness and reformulate negative thoughts seems to be important in this case. In a study by Schou and Ekeberg, it was shown that patients with breast cancer and internal locus of health control used adaptive coping styles to deal with stress [39]. Similar results to ours were presented by Lisowska and Slowik-Gabrylewska, investigating the locus of health control and quality of life in patients with ovarian cancer. It was shown that internal locus of control of life was correlated with higher quality of life and patients’ adoption of adaptive coping styles [40].

A psycho-oncological intervention can lead to a change in patients attitudes regarding their sense of control over their health and management of their stress coping styles. In our research, we showed that female patients who were convinced that they were making personal efforts to fight their diseases felt more confident in their own efforts. Self-efficacy was higher in patients whose locus of control over their health was dependent on the influence of others. These patients may redirect their attention to the actions of the medical team, which is responsible for the planning and conduct of the treatment process. As we have shown, these actions result in an increased sense of self-efficacy in female patients. Similarly, in a study by Náfrádi and others, it was found that patients who placed external control on doctors were more likely to follow their recommendations and guidelines [41]. There are no studies in the literature on the location of health control and self-efficacy in women with ovarian cancer. A number of studies on other cancers indicate that patients with higher self-efficacy tend to locate health control internally more often [42,43,44].

The use of screening tools based on the example of The National Comprehensive Cancer Network (NCCN) can be helpful in detecting distress in patients with ovarian cancer in sexual, physiological, emotional and family areas. The use of such tools allows the monitoring of treatment side effects, and contributes to improve and organize life with and after cancer [45]. Adequate communication with patients has an important role in enhancing a sense of meaning in life and quality of life [46]. Psychological interventions and meaning-focused psychotherapy are indicated to be effective in improving patients’ quality of life and meaning of life. The implementation of interventional psychoeducation for female patients contributes to improvements in sexual desire, arousal and the orgasmic state experience. A lower incidence of depressive symptoms and sexual anxiety is also observed among patients submitted to cognitive therapy combined with education [47]. The use of screening tools to determine the level of distress in female patients represents only half success. Adequate communication between doctor and patient, as well as among medical staff and the patient’s family, plays an essential role in recovering the psychological balance of female patients.

## 5. Conclusions

For patients, adequate communication with the medical team is especially important throughout the treatment process. Adequate communication during initial diagnosis and treatment can reduce patients’ anxiety related to a lack of information about the disease [48]. Our study indicates the need for patients to receive ongoing psychological support during treatment. Maladaptive coping styles predispose patients to higher levels of illness. These patients also tend to place control externally and have lower levels of self-efficacy [49]. The role of the psychologist and psycho-oncologist must focus not only on alleviating the crises experienced by female patients but also to reformulate maladaptive forms of behaviour. Incorporating psychological surveys measuring both coping styles, self-efficacy and locus of health control into the care of patients with ovarian cancer can contribute to more effective psychological intervention which will benefit the patient’s quality of life.

## 6. Limitations

The study had several limitations. The first was the limited study sample; however, collecting a larger group of patients with ovarian cancer would have significantly prolonged the study time, which is why we decided to terminate the study after reaching the minimum number of patients we had set. In our study, we focused only on ovarian cancer, which, on the one hand, can be considered as a limitation, but on the other hand, there are still very few studies in the literature dedicated to this type of cancer. Given the low 5-year survival rate we found, and the range of psychological difficulties faced by patients, we decided to study only this cancer. A last limitation of the study was the use of cross-sectional studies.

## Figures and Tables

**Table 1:** Basic descriptive statistics of quantitative variables.

	*M*	*Me*	*SD*	*Min.*	*Max.*	*D*	*p*
Task-focused style	53.13	53	9.12	31	75	0.05	0.200
Emotion-focused style	41.95	43	9.48	16	63	0.09	0.018
Avoidance-focused style	45.97	47	7.60	29	63	0.09	0.011
Engaging in vicarious activities	21.50	22	5.25	9	38	0.13	<0.001
Seeking social contact	15.87	16	3.58	7	24	0.10	0.004
Internal control (A)	22.98	23	5.02	11	35	0.06	0.200
External control (A)	23.97	24	5.19	10	36	0.08	0.025
Random control (A)	22.34	23	5.42	6	36	0.07	0.086
Self-efficacy	28.71	29	4.73	15	38	0.09	<0.001

*M*—average; *Me*—mediana; *SD*—standard deviation; *Min.* and *Max.*—lowest and highest values of the distribution; *D*—Kolmogorov-Smirnov test result; *p*—relevance.

**Table 2:** The level of self-efficacy and the level of styles of coping with stress.

		Self-Efficacy
Task-focused style	Pearson’s r	0.50
	*p*-value	<0.001
Emotion-focused style	Pearson’s r	−0.38
	*p*-value	<0.001
Avoidance-focused style	Pearson’s r	−0.08
	*p*-value	0.388
Engaging in vicarious activities	Pearson’s r	−0.24
	*p*-value	0.007
Seeking social contact	Pearson’s r	0.23
	*p*-value	0.008

**Table 3:** The level of health locus of control (variant A) and the level of styles of coping with stress.

		Internal Control	External Control	Random Control
Task-focused style	Pearson’s r	0.16	0.19	−0.01
	*p*-value	0.077	0.035	0.880
Emotion-focused style	Pearson’s r	0.00	−0.12	0.25
	*p*-value	0.989	0.172	0.005
Avoidance-focused style	Pearson’s r	0.01	−0.07	−0.14
	*p*-value	0.935	0.453	0.130
Engaging in vicarious activities	Pearson’s r	0.01	−0.09	−0.10
	*p*-value	0.932	0.291	0.242
Seeking social contact	Pearson’s r	−0.01	0.04	−0.07
	*p*-value	0.934	0.673	0.439

**Table 4:** The level of health locus of control (variant A) and the level of self-efficacy.

		Self-Efficacy
Internal control	Pearson’s r	0.28
	*p*-value	<0.001
External control	Pearson’s r	0.21
	*p*-value	0.015
Radom control	Pearson’s r	0.11
	*p*-value	0.199

## Data Availability

The data presented in this study are available on request from the first author.

## Supplementary Materials

The following supporting information can be downloaded at: https://www.mdpi.com/article/10.3390/jcm12216816/s1, Questionnaire Q1: the General Self-Efficacy Scale (GSES). Questionnaire Q2: the Multidimensional Health Locus of Control. Questionnaire Q3: the Coping Inventory for Stressful Situations (CISS).

## References

[1] Jayson G.C., Kohn E.C., Kitchener H.C., Ledermann J.A. (2014). Ovarian cancer. Lancet.

[2] Bregenzer M.E., Horst E.N., Mehta P., Novak C.M., Repetto T., Mehta G. (2019). The Role of Cancer Stem Cells and Mechanical Forces in Ovarian Cancer Metastasis. Cancers.

[3] Schwarzer R., Boehmer S., Luszczynska A., Mohamed N.E., Knoll N. (2005). Dispositional self-efficacy as a personal resource factor in coping after surgery. Personal. Individ. Differ..

[4] Simard S., Savard J., Ivers H. (2010). Fear of cancer recurrence: Specific profiles and nature of intrusive thoughts. J. Cancer Surviv..

[5] Schneider-Matyka D., Skwirczyńska E., Gaur M., Hukowska-Szematowicz B., Kwiatkowski S., Mikla M., Grochans E., Cymbaluk-Płoska A. (2022). Evaluation of the Influence of Biological Factors during the Course of Treatment in Patients with Ovarian Cancer. Int. J. Environ. Res. Public Health.

[6] Herschbach P., Book K., Dinkel A., Berg P., Waadt S., Duran G., Engst-Hastreiter U., Henrich G. (2010). Evaluation of two group therapies to reduce fear of progression in cancer patients. Cancer.

[7] Bandura A. (1977). Self-efficacy. Toward a unifying theory of behavioral change. Psychol. Rev..

[8] Schwarzer R., Fuchs R. (1996). Self-Efficacy and Health Behaviours. Predicting Health Behavior: Research and Practice with Social Cognition Models.

[9] Malicka I., Szczepańska J., Anioł K., Rymaszewska J., Woźniecki M. (2009). Zaburzenia nastroju strategie przystosowania do choroby u kobiet leczonych operacyjnie z powodu nowotworu piersi i narządów rodnych. Współcz. Onkol..

[10] Cunningham A.J., Lockwood G.A., Cunningham J.A. (1991). A relationship between perceived self-efficacy and quality of life in cancer patients. Patient Educ. Couns..

[11] Wallston K.A., Wallston B.S., DeVellis R. (1978). Development of the Multidimensional Health Locus of Control (MHLC) Scales. Health Educ. Monogr..

[12] Meadows R.J., Nolan T.S., Paxton R.J. (2019). Spiritual health locus of control and life satisfaction among African American breast cancer survivors. J. Psychosoc. Oncol..

[13] Gibek K., Sacha T. (2019). Comparison of health locus of control in oncological and non-oncological patients. Współcz. Onkol..

[14] Hashimoto H., Fukuhara S. (2004). The influence of locus of control on preferences for information and decision making. Patient Educ. Couns..

[15] BorjAlilu S., Kaviani A., Helmi S., Karbakhsh M., Mazaheri M.A. (2017). Exploring the role of self-efficacy for coping with breast cancer: A systematic review. Arch. Breast Cancer.

[16] Topcu S., Oguz S. (2017). Self-efficacy and quality of life after stroke. J. Hum. Sci..

[17] Basińska M., Zalewska-Rydzkowska D., Wolańska P., Junik R. (2008). Dyspozycyjny optymizm a akceptacja choroby w grupie osób z chorobą Gravesa-Basedowa. Pol. J. Endocrinol..

[18] Heszen-Niejodek I., Gruszczyńska E. (2004). Wymiar duchowy człowieka, jego znaczenie w psychologii zdrowia i jego pomiar. Prz. Psychol..

[19] Borhani F., Abbas-Zadeh A., Taebi M., Kohan S. (2010). The relationship between personal efficiency and health beliefs of patients with Type 2 diabetes’ (Persian). Payesh Q..

[20] Endler N.S., Parker J.D.A. (1994). Assessment of multidimensional coping: Task, emotion, and avoidance strategies. Psychol. Assess..

[21] Lazarus R.S., Folkman S. (1987). Transactional theory and research on emotions and coping. Eur. J. Personal..

[22] Vargas-Román K., Tovar-Gálvez M.I., Liñán-González A., Cañadas de la Fuente G.A., de la Fuente-Solana E.I., Díaz-Rodríguez L. (2022). Coping Strategies in Elderly Colorectal Cancer Patients. Eur. J. Cancers.

[23] Carver C.S., Scheier M.F., Weintraub J.K. (1989). Assessing coping strategies: A theoretically based approach. J. Personal. Soc. Psychol..

[24] Arraras J.I., Wright S.J., Jusue G., Tejedor M., Calvo J.I. (2002). Coping style, locus of control, psychological distress and pain-related behaviours in cancer and other diseases. J. Psychol. Health Med..

[25] Moreno-Smith M., Lutgendorf S.K., Sood A.K. (2010). Impact of stress on cancer metastasis. Future Oncol..

[26] Doumit M.A., Huijer H.A., Kelley J.H., El Saghir N., Nassar N. (2010). Coping with breast cancer: A phenomenological study. Cancer Nurs..

[27] Culver J.L., Arena P.L., Antoni M.H., Carver C.S. (2002). Coping and distress among women under treatment for early stage breast cancer: Comparing African Americans, Hispanics and Whites. Psychooncology.

[28] Lindau S.T., Gavrilova N. (2010). Sex, health, and years of sexually active life gained due to good health: Evidence from two US population based cross sectional surveys of ageing. BMJ.

[29] Chida Y., Hamer M., Wardle J., Steptoe A. (2008). Do stress-related psychosocial factors contribute to cancer incidence and survival?. Nat. Clin. Pract. Oncol..

[30] Juczyński Z. (2000). Poczucie Własnej Skuteczności–Teoria i Pomiar.

[31] Strelau J., Jaworowska A., Wrześniewski K., Szczepaniak P. (2009). CISS Kwestionariusz Radzenia Sobie w Sytuacjach Stresowych: Kwestionariusz Radzenia Sobie w Sytuacjach Stresowych Podręcznik do Polskiej Normalizacji.

[32] George D., Mallery M. (2019). IBM SPSS Statistic 25 Step by Step: A Simple Guide and Reference.

[33] Bakan G., Inci F.H. (2021). Predictor of self-efficacy in individuals with chronic disease: Stress-coping strategies. J. Clin. Nurs..

[34] Chirico A., Lucidi F., Merluzzi T., Alivernini F., Laurentiis M., Botti G., Giordano A. (2017). A meta-analytic review of the relationship of cancer coping self-efficacy with distress and quality of life. Oncotarget.

[35] Kreitler S., Peleg D., Ehrenfeld M. (2007). Stress, self-efficacy and quality of life in cancer patients. Psycho-Oncology.

[36] Amirshamsi M., Shahrbabaki P.M., Dehghan M. (2022). Coping strategies for stress and self-efficacy in patients with cancer and their spouses: A cross-sectional study. Cancer Nurs..

[37] Lang-Rollin I., Berberich G. (2018). Psycho-oncology. Dialogues Clin. Neurosci..

[38] Merluzzi T.V., Pustejovsky J.E., Philip E.J., Sohl S.J., Berendsen M., Salsman J.M. (2019). Interventions to enhance self-efficacy in cancer patients: A meta-analysis of randomized controlled trials. Psycho-Oncology.

[39] Schou I., Ekeberg Ø. (2000). The role of spirituality and religious coping in the quality of life of patients with advanced cancer receiving palliative radiation therapy. J. Psychosoc. Oncol..

[40] Lisowska K., Słowik-Gabryelska A. (2017). Styl radzenia sobie ze stresem, umiejscowienie kontroli zdrowia i jakość życia u pacjentek z rakiem jajnika. Pielęgniarstwo Zdr. Publiczne.

[41] Náfrádi L., Nakamoto K., Schulz P.J. (2017). Is patient empowerment the key to promote adherence? A systematic review of the relationship between self-efficacy, health locus of control and medication adherence. PLoS ONE.

[42] Keinki C., Seilacher E., Ebel M., Ruetters D., Kessler I., Stellamanns J., Rudolph I., Huebner J. (2016). Information needs of cancer patients and perception of impact of the disease, of self-efficacy, and locus of control. J. Cancer Educ..

[43] Wang R., Zhou C., Wu Y., Sun M., Yang L., Ye X., Zhang M. (2022). Patient empowerment and self-management behaviour of chronic disease patients: A moderated mediation model of self-efficacy and health locus of control. J. Adv. Nurs..

[44] Welter S., Keinki C., Ahmadi E., Huebner J. (2021). Lay etiology, self-efficacy and patient activation among cancer patients. Cancer Investig..

[45] Hasenburg A., Sehouli J. (2022). Fotopoulou CPeri-operative ovarian cancer guidelines: Psycho-oncologyInternational. J. Gynecol. Cancer.

[46] Teo I., Krishnan A., Lee G.L. (2019). Psychosocial interventions for advanced cancer patients: A systematic review. Psycho-Oncology.

[47] Whicker M., Black J., Altwerger G., Menderes G., Feinberg J., Ratner E. (2017). Management of sexuality, intimacy, and menopause symptoms in patients with ovarian cancer. Am. J. Obstet. Gynecol..

[48] Rietveld M.J.A., Husson O., Vos M.C.C., van de Poll-Franse L.V., Ottevanger P.B.N., Ezendam N.P.M. (2018). Association between information provision and supportive care needs among ovarian cancer survivors: A cross-sectional study from the PROFILES registry. Psycho-Oncology.

[49] Roddenberry A., Renk K. (2010). Locus of Control and Self-Efficacy: Potential Mediators of Stress, Illness, and Utilization of Health Services in College Students. Child Psychiatry Hum. Dev..

