# Supplementary material for: Psychological Associations of Stress with the Level of Health Locus of Control and Self-Efficacy in Patients with Ovarian Cancer
Source: J Clin Med. 2023 Oct 28;12(21):6816. doi: 10.3390/jcm12216816 (PMC10650488; doi:10.3390/jcm12216816)
Supplement: Supplementary file 1 [file jcm-12-06816-s001.zip › jcm-2541384-supplementary.pdf]

Q1

R. Schwarzer, M. Jerusalem, Z. Juczyński

Wersja polska GSES

Wydanie drugie

.... wiek................płeć M K data badania........................

Poniżej przedstawiono kilka stwierdzeń odnoszących się do różnych cech osobistych. Po przeczytaniu każdego stwierdzenia należy zdecydować, czy w stosunku do ciebie są one prawdziwe czy fałszywe.

Poszczególne punkty skali oznaczają:

1 – NIE **2** – raczej NIE **3** – raczej TAK **4** – TAK

---

1. Zawsze jestem w stanie rozwiązać trudne problemy, jeśli tylko wystarczająco się postaram 1 2 3 4
2. Nawet, gdy ktoś mi się sprzeciwia, jestem w stanie znaleźć sposób na osiągnięcie tego, czego chcę 1 2 3 4
3. Z łatwością potrafię trzymać się swoich celów i je osiągać 1 2 3 4
4. Jestem przekonany, że skutecznie poradziłbym sobie z nieoczekiwanymi wydarzeniami 1 2 3 4
5. Dzięki swojej pomysłowości i zaradności wiem, jak poradzić sobie z nieprzewidzianymi sytuacjami 1 2 3 4
6. Jestem w stanie rozwiązać większość problemów, jeśli tylko włożę w to odpowiednio dużo wysiłku 1 2 3 4
7. Kiedy zmagam się z przeciwnościami, jestem w stanie zachować spokój, gdyż mogę polegać na swoich umiejętnościach radzenia sobie . .1 2 3 4
8. Kiedy zmagam się z jakimś problemem, to zazwyczaj jestem w stanie znaleźć kilka sposobów jego rozwiązania 1 2 3 4
9. Gdy mam kłopoty, to zazwyczaj jestem w stanie wymyślić sposób, jak z nich wyjść 1 2 3 4
10. Zazwyczaj jestem w stanie poradzić sobie z tym, co mnie spotyka 1 2 3 4

PWS	sten

Wydanie polskie: Copyright © 2012 by Pracownia Testów Psychologicznych Polskiego Towarzystwa Psychologicznego Sp. z o.o.
ul. Belwederska 6A, 00-762 Warszawa
www.practest.com.pl

Q2

K. A. Wallston, B. S .Wallston i R. DeVellis

MHLC - wersja A

Adaptacja: Z. Juczyński

.... wiek .... płeć M K data badania ....

Skala przedstawia poglądy różnych ludzi na pewne istotne zagadnienia związane ze zdrowiem. Każde stwierdzenie wyraża pogląd, z którym można się zgodzić lub nie. Chodzi tu o osobiste przekonanie i nie ma tu odpowiedzi ani dobrych ani złych. Ważne jest, aby odpowiadać zgodnie z własnymi przekonaniami, a nie z tym – co powinno się sądzić.

Obok znajdują się odpowiedzi od ***„zdecydowanie nie zgadzam się”*** (1) – do ***„zdecydowanie zgadzam się”*** (6). Przy każdym zdaniu należy otoczyć kółkiem liczbę, która wyraża właściwy stopień zgody. Należy podać jedną odpowiedź dla każdego stwierdzenia. Poszczególne liczby oznaczają:

**NIE** zdecydowanie nie zgadzam się **1**	w pewnym stopniu nie zgadzam się **2**	w małym stopniu nie zgadzam się **3**	w małym stopniu zgadzam się **4**	w pewnym stopniu zgadzam się **5**	**TAK** zdecydowanie zgadzam się **6**

1. Kiedy choruję, to od mojego zachowania zależy, jak szybko wyzdrowieję 1 2 3 4 5 6
2. Jeżeli mam zachorować, to zachoruję niezależnie od tego, co zrobię 1 2 3 4 5 6
3. Utrzymywanie regularnych kontaktów z lekarzem jest najlepszym sposobem uniknięcia choroby 1 2 3 4 5 6
4. Większość tego, co wpływa na moje zdrowie, zdarza się przez przypadek 1 2 3 4 5 6
5. Kiedy tylko poczuję się źle, konsultuję się z lekarzem 1 2 3 4 5 6
6. Ja sam decyduję o swoim zdrowiu 1 2 3 4 5 6
7. Inni ludzie (np. lekarze, pielęgniarki, rodzina, przyjaciele) mają wpływ na to, czy zachoruję, czy pozostanę zdrowy 1 2 3 4 5 6
8. Kiedy choruję, wina leży po mojej stronie 1 2 3 4 5 6
9. To, jak szybko wyzdrowieję, zależy w dużym stopniu od szczęścia 1 2 3 4 5 6
10. Lekarze decydują o moim zdrowiu 1 2 3 4 5 6

Proszę odwrócić kartkę

Wydanie polskie: Copyright © 2012 by Pracownia Testów Psychologicznych Polskiego Towarzystwa Psychologicznego Sp. z o.o.
ul. Belwederska 6A, 00-762 Warszawa
www.practest.com.pl

NIE zdecydowanie nie zgadzam się 1	w pewnym stopniu nie zgadzam się 2	w małym stopniu nie zgadzam się 3	w małym stopniu zgadzam się 4	w pewnym stopniu zgadzam się 5	TAK zdecydowanie zgadzam się 6

11. Kiedy jestem zdrowy, mam po prostu szczęście 1 2 3 4 5 6
12. Moje samopoczucie fizyczne zależy od tego, jak dobrze dbam o siebie . . . 1 2 3 4 5 6
13. Kiedy choruję, to wiem, że to dlatego, iż o siebie nie zadbałem 1 2 3 4 5 6
14. Opieka, którą otrzymuję od innych, decyduje o tym jak szybko powracam do zdrowia 1 2 3 4 5 6
15. Nawet jeśli dbam o siebie, łatwo mogę zachorować 1 2 3 4 5 6
16. Kiedy choruję, jest to sprawa losu 1 2 3 4 5 6
17. Pozostanę raczej zdrowy, jeżeli dobrze zadbam o siebie 1 2 3 4 5 6
18. Dokładne wypełnianie poleceń lekarza jest najlepszym sposobem zachowania dobrego zdrowia 1 2 3 4 5 6

W	I	P

Q3

KWESTIONARIUSZ CISS

Norman S. Endler & James D. A. Parker

Imię i nazwisko .... Wiek .... Płeć ....

Wykształcenie .... Zawód .... Data badania ....

INSTRUKCJA: poniższe zadania opisują różne reakcje ludzi na trudne, przykre, stresujące sytuacje. Zakreśl kółkiem jedną z cyfr od 1 do 5 przy każdym stwierdzeniu. Określ w ten sposób, jak bardzo angażujesz się w te czynności, gdy znajdziesz się w trudnej, przykrej, stresującej sytuacji.

1 - nigdy
2 - bardzo rzadko
3 - czasami
4 - często
5 - bardzo często

1. Lepiej planuję swój czas 1 2 3 4 5
2. Koncentruję się na problemie i zastanawiam się, jak mogę go rozwiązać 1 2 3 4 5
3. Myślę o czasach gdy było mi lepiej 1 2 3 4 5
4. Staram się przebywać z innymi ludźmi 1 2 3 4 5
5. Oskarżam się o zwlekanie 1 2 3 4 5
6. Robię to, co uważam za najlepsze 1 2 3 4 5
7. Jestem skupiony(a) na swoich dolegliwościach fizycznych 1 2 3 4 5
8. Winię siebie, że wpadłem(am) w taką sytuację 1 2 3 4 5
9. Włóczę się po sklepach 1 2 3 4 5
10. Ustalam, co w danej sytuacji jest najważniejsze 1 2 3 4 5
11. Staram się zasnąć 1 2 3 4 5
12. Objadam się ulubioną potrawą 1 2 3 4 5
13. Niepokoję się, że sobie nie poradzę 1 2 3 4 5
14. Staję się bardzo napięty(a) 1 2 3 4 5
15. Myślę o tym, jak rozwiązywałem(am) podobne problemy w przeszłości 1 2 3 4 5
16. Wmawiam sobie, że to w rzeczywistości nie dzieje się mnie 1 2 3 4 5
17. Winię siebie, że zbyt się tym przejmuję 1 2 3 4 5
18. Idę coś zjeść na mieście 1 2 3 4 5

Copyright © [1999], Multi-Health Systems Inc. Międzynarodowe prawo autorskie obowiązujące we wszystkich krajach w ramach Konwencji z Berna, Bilateralnej Konwencji o Prawie Autorskim i Uniwersalnej Konwencji o Prawie Autorskim. Wszystkie prawa zastrzeżone. Zabrania się dokonywania tłumaczenia lub powielania w całości lub w części, przechowywania w jakichkolwiek bankach danych oraz przesyłania w jakiejkolwiek formie lub jakimikolwiek środkami kopiującymi, mechanicznymi, elektronicznymi, zapisującymi lub innymi, bez wcześniejszej pisemnej zgody Multi-Health Systems Inc., 3770 Victoria Park Avenue, Toronto, Ontario M2H 3M6. Polskie wydanie zaadaptowane i opublikowane w **2003** roku przez **Pracownię Testów Psychologicznych Polskiego Towarzystwa Psychologicznego**, ul. Belwederska 6A, 00-762 Warszawa, na mocy licencji udzielonej przez Multi-Health Systems Inc.

19. Staję się bardzo przygnębiony(a) ... 1 2 3 4 5
20. Kupuję sobie coś ... 1 2 3 4 5
21. Wyznaczam sobie kierunek działania i postępuję zgodnie z nim ... 1 2 3 4 5
22. Obwiniam siebie za to, że nie wiem co zrobić ... 1 2 3 4 5
23. Idę się zabawić ... 1 2 3 4 5
24. Staram się zrozumieć sytuację ... 1 2 3 4 5
25. "Zastygam w bezruchu" i nie wiem co zrobić ... 1 2 3 4 5
26. Podejmuję natychmiast właściwe działanie ... 1 2 3 4 5
27. Analizuję sytuację i uczę się na własnych błędach ... 1 2 3 4 5
28. Żałuję, że nie mogę zmienić tego co się stało, lub tego, co odczuwałem(am) w związku z tym ... 1 2 3 4 5
29. Odwiedzam przyjaciela ... 1 2 3 4 5
30. Martwię się, jak sobie z tym poradzę ... 1 2 3 4 5
31. Spędzam czas z bliską osobą ... 1 2 3 4 5
32. Wychodzę na spacer ... 1 2 3 4 5
33. Wmawiam sobie, że to się nigdy więcej nie powtórzy ... 1 2 3 4 5
34. Skupiam się na swoich ogólnych brakach ... 1 2 3 4 5
35. Rozmawiam z kimś, kogo rady sobie cenię ... 1 2 3 4 5
36. Analizuję problem zanim zacznę działać ... 1 2 3 4 5
37. Dzwonię do kolegi lub koleżanki ... 1 2 3 4 5
38. Wpadam w złość ... 1 2 3 4 5
39. Zmieniam kolejność spraw do załatwienia ... 1 2 3 4 5
40. Oglądam film ... 1 2 3 4 5
41. Dążę do kontrolowania sytuacji ... 1 2 3 4 5
42. Podejmuję dodatkowy wysiłek, aby załatwić sprawę ... 1 2 3 4 5
43. Podchodzę do problemu z różnych stron ... 1 2 3 4 5
44. "Robię sobie wolne", by uciec od problemu ... 1 2 3 4 5
45. Wyładowuję się na innych ... 1 2 3 4 5
46. Wykorzystuję sytuację, aby udowodnić, że potrafię tego dokonać ... 1 2 3 4 5
47. Staram się tak zorganizować sprawy, aby zapanować nad sytuacją ... 1 2 3 4 5
48. Oglądam telewizję ... 1 2 3 4 5

Sprawdź, czy ustosunkowałeś się do wszystkich stwierdzeń !

Copyright © [1999], Multi-Health Systems Inc. Międzynarodowe prawo autorskie obowiązujące we wszystkich krajach w ramach Konwencji z Berna, Bilateralnej Konwencji o Prawie Autorskim i Uniwersalnej Konwencji o Prawie Autorskim. Wszystkie prawa zastrzeżone. Zabrania się dokonywania tłumaczenia lub powielania w całości lub w części, przechowywania w jakichkolwiek bankach danych oraz przesyłania w jakiejkolwiek formie lub jakimikolwiek środkami kopiującymi, mechanicznymi, elektronicznymi, zapisującymi lub innymi, bez wcześniejszej pisemnej zgody Multi-Health Systems Inc., 3770 Victoria Park Avenue, Toronto, Ontario M2H 3M6. Polskie wydanie zaadaptowane i opublikowane w **2003** roku przez **Pracownię Testów Psychologicznych Polskiego Towarzystwa Psychologicznego**, ul. Belwederska 6A, 00-762 Warszawa, na mocy licencji udzielonej przez Multi-Health Systems Inc.
